# In-depth Biological Monitoring to Estimate Effects of Red or Processed Meat on Colorectal Cancer

**DOI:** 10.7150/jca.126466

**Published:** 2026-01-14

**Authors:** Joon Seok Lee, Eunbee Kim, Un Jae Lee, Myung-Ah Lee, Ae-son Um, Hyun-Shik Lee, Mihi Yang

**Affiliations:** 1 Agilent Technologies Korea, Seoul, Republic of Korea.; 2 School of Life Sciences, College of Natural Science, Kyungpook National University, Daegu, Republic of Korea.; 3 Department of Toxicology, College of Pharmacy, Sookmyung Women's University, Seoul, Republic of Korea.; 4 Goodbeing Co., Ltd., Seoul, Republic of Korea.; 5 Department of Internal Medicine, Seoul St. Mary's Hospital, College of Medicine, The Catholic University of Korea, Seoul, Republic of Korea.; 6 Department of Food and Nutrition, College of Human Ecology, Hanyang University, Seoul, Republic of Korea.

**Keywords:** colorectal cancer, biological monitoring, red meat, processed meat, oxidative stress, DNA-adducts

## Abstract

**Background**: The intake of red or processed meat remains controversial as a crucial factor for CRC. Thus, we performed in-depth biological monitoring.

**Methods:** We performed a case- control study and analyzed various exposure and response biomarkers including 1-OHP, MeIQx, and PhIP, and malondialdehyde (MDA), and heterocyclic amine (HCA)-DNA adducts in Korean cases and controls (N = 218).

**Results:** They consumed 53.4 ± 74.0 g/day of red meat and 1.1 ± 3.7 g/day of processed meat. The CRC presence was associated with sex, BMI, tobacco smoking, alcohol drinking, cooking method of meat, and vegetable consumption, rather than red or processed meat intake. The levels of MDA were positively associated with those of 1-OHP, MeIQx, or PhIP. The sum of 1-OHP, MeIQx, and PhIP was associated with the levels of HCA-DNA adducts and cooking method of meat. In addition, the above biomarkers for CRC were associated with each other. However, most of these biomarkers were not higher in CRC patients than those in controls.

**Conclusion:** The present in-depth biological monitoring provides that red or processed meat may induce oxidative stress; however, present intake of the meat and the intake-related oxidative stress may not affect CRC prevalence among the Korean population, who consume less meat than Westerners.

## Introduction

The global burden of colorectal cancer (CRC) is estimated to be more than 3.2 million new cases per year by 63% from 2020 to 2040 1. However, there are big differences in the incidences of CRC among the countries. For example, the incidence rate of CRC in USA was 36.9 for 2017-2021 and 27.2 per 100, 000 persons in South Korea for 2020 2-3. Therefore, CRC disparity may be affected by different eating habits for each ethnic group. Because of high potential of prevention from foodborne carcinogens, CRC has been emphasized as an avoidable cancer and biological monitoring with precise biomarkers for the carcinogens can be a good approach for precision prevention from CRC.

Traditionally, biological monitoring has been performed for environmental and occupational health. However, the use of proper biomarkers has expanded the scope of biological monitoring to precision prevention and medicine for exposure-related diseases 4. Red meat and processed meat have been designated as 'probably carcinogenic to humans' (Group 2A) and 'carcinogenic to humans' (Group 1), respectively. The International Agency for Research on Cancer declared that each 50 g of processed meat and each 100 g of red meat eaten daily increase the risk of CRC by 18% and 17%, respectively 5. Thus, growing consumption of red meat and processed meat might increase CRC in newly industrializing countries. However, our pilot study in South Korea has shown that other factors rather than amounts of meat affected CRC 6. For example, lipid metabolites, such as total fatty acids, saturated fatty acids, or polyunsaturated fatty acids were approx. 2-fold higher in CRC cases and controls 6.

In addition, the amount of red meat and processed meat is lower in South Korea than in western countries, such as USA, UK, and so on 7. Thus, whether the intake of red meat and processed meat is a crucial factor for CRC in Koreans remains controversial 8.

To address the meat intake issue, regulatory agencies and toxicologists need more evidence-based methods, such as biological monitoring with specific exposure biomarkers for the meat. For this purpose, we focused on exposure to polycyclic aromatic hydrocarbons (PAHs) and heterocyclic amines (HCAs) known to be produced during cooking of meats at high temperatures (≥ 150 ºC) and analyzed biomarkers for their exposure, such as 1-hydroypyrnene (OHP) for PAHs 9 and 2-Amino-3,8-dimethyl-3H-imidazo (4,5-j) quinoxaline (MeIQx) and 2-Amino-1-methyl-6-phenylimidazo[4,5-b] pyridine (PhIP) for HCAs 6, 10. Biological monitoring can also provide carcinogenic mechanisms between causes and diseases, i.e., carcinogens and a certain cancer. For example, reactive oxygen species (ROS) can be involved in toxic or carcinogenic mechanisms of CRC 11. Response biomarkers for ROS, malondialdehyde (MDA) or C-reactive protein (CRP), can be carcinogenic biomarkers caused by the lipid peroxidation pathway 12. In addition, DNA-adducts of HCAs as bio-produced carcinogens by high temperature cooking of red meat can provide longitudinal exposure and carcinogenic evidences of red meat and processed meat for CRC.

Thus, we performed enlarged case-control study with biological monitoring, based on our previous pilot study 6. In order to clarify crucial factors for effects of red meat and processed meat consumption on CRC, we focused on a Korean population, who consume less meat than Westerners.

## Materials and Methods

### Study design

The sample size was calculated from differences in urinary PhIP levels between cases and controls, which was the middle sample size among the calculated numbers from various biomarkers (power = 0.8; α = 0.05). It was estimated that 98 subjects in a group would be needed. Considering a 10% of dropout rate (≥ 10 person/group), thus, we recruited 218 subjects during 2017-18. This study was conducted in accordance with the Declaration of Helsinki and approved by Seoul St. Mary's Hospital, the Catholic Medical Center, Seoul, Korea (IRB#, KC18QNSI0057) for all of study procedures and contents including human ethics and informed consents. Fig. S1 and Table S1 show 'inclusion and exclusion criteria' and enrollment requirements in detail. In brief, we included the newly diagnosed cases within 2 weeks with CRC. To avoid any kind of food intervention, we excluded the people who currently changed their life style including food. Primary end point was urinary PhIP and second end points were various other exposure or response biomarkers for the meat.

Written informed consent was obtained from each study participants prior to inclusion in this study. Newly diagnosed CRC patients and heathy controls were recruited from people, who visited the medical center for regular examination (116 men and 102 women; mean age: 64.44 ± 13.41 years). We excluded those with a previous cancer, those with a history of colorectal polyps, those with a history of inflammatory disease, and those with a family history of hereditary CRC.

When subjects were enrolled, we collected 40 ml of urine and 13 ml of peripheral blood from the subjects. The blood samples were collected into three different tubes, including 5 ml in an EDTA tube, 5 ml in a clot activator gel tube. After EDTA blood tube samples were centrifuged at 14,000 rpm for 10 mins at 4 ºC, buffy coats and plasma fraction were separated and stored at -20 ºC before experiments. The peripheral blood in the clot activator gel tube was centrifuged at the same condition of the above. The separated serum fraction was used to analyze hematological indicators, such as aspartate transaminase (AST), alanine aminotransferase (ALT), CRP, total cholesterol (TC), triglyceride (TG), low density lipoprotein (LDL-cholesterol), high density lipoprotein (HDL-cholesterol), and homocysteine with an automatic biomedical analyzer (HITACHI 7020, Tokyo, Japan). Urinary creatinine levels were also analyzed with the automatic analyzer.

All subjects were interviewed to fill out a food frequency questionnaire (FFQ) to evaluate food intake a year before diagnosis and a lifestyle questionnaire, which was developed by us to study lifestyle including tobacco, alcohol, degree of cooked meat, intake of meat, fruits, or vegetables, exercise, etc. The FFQ was also used to calculate dietary inflammatory index (DII) score, a literature-derived and population-based dietary scoring system (DII^®^).

### Exposure assessment

Urinary 1-hydroxypyrene (1-OHP) was measured to monitor red or processed meat-induced PAHs with a reverse phase HPLC/FD method 6. In detail, 200 µl of urine was hydrolyzed with 30 µL of β-glucuronidase (2,550 units; Sigma-Aldrich, St. Louis, MO, USA) after addition of 200 ul of 0.2 M sodium acetate buffer (pH 5.0) followed by incubation for more than 5 hours at 37°C. After the incubation, 570 µl of acetonitrile (ACN) was added to the mixture followed by centrifugation at 14,000 rpm for 10 min. The supernatant of the mixture was transferred to an HPLC vial. The HPLC system consisted of a YL9111 binary pump (Yonglin Co., Seoul, Korea), a YL9150 auto-sampler (Yonglin Co.), a Jasco FP-2020 plus FD (Jasco, Tokyo, Japan), and a YMC-Triart C18 column (150mm x 4.6mm, 3.0um; YMC Co LTD., Kyoto, Japan). The mobile phase was 65% ACN in water. Excitation and emission wavelengths were 242 nm and 388 nm, respectively.

For urinary HCAs, we performed liquid-liquid extraction following our previous method 6 with an ultra high performance liquid chromatography with tandem mass spectrometry (UPLC/MS/MS) system and analyzed two biomarkers, MeIQx and PhIP. Briefly, 2 ml of urine was mixed with 4 µl of internal standards, i.e., 0.57 µM of MeIQx-d_3_ and 0.26 µM of PhIP-d_3_ (Toronto Research Chemicals, North York, ON, Canada) and hydrolyzed with 200 ul of 10 N NaOH. The mixture was twice extracted with CH_2_Cl_2_. The extract was dried in a SpeedVac concentrator (Savant Inc., Farmingdale, NY, USA) and dissolved in 100 ul of 50% of ACN. After centrifuging, the supernatant was transferred to a vial of LC/MS/MS compatible with an Agilent 1260 Infinity UPLC system (Agilent Technologies, Santa Clara, CA) and MS, an Agilent Triple Quadrupole 6460 system with a specialized type of ESI interface. The mobile phase was a binary mixture of 0.01% of formic acid and 20 mM of ammonium formate in water (A) and ACN (B). These two mobile phases were used in a gradient mode at a flow rate of 0.4 ml/min. Gradient conditions were: 5% of B for 1 min, increasing B to 95% for 8 min with a linear gradient, retaining 95% of B for 2 min to wash the column, YMC Meteoric Core C18 (50 x 3.0 mm i.d., 2.7 um particle size), and decreasing B to 5% for 3 min. Five 5 µl of the supernatant was injected into the UPLC system. The column temperature was maintained at 35°C. The MS conditions and calicuration curves of HCAs are in supplements as Table S2, Fig. S2 and Fig. S3, respectively.

### Response assessment

We analyzed MDA for oxidative stress in urine with some modification of our previous prepation method 6, using a more sensitive reverse phase HPLC with fluorescence ditector than the previous method with UV ditector.

We also analyzed two major HCA-DNA adducts, dG-C8-MeIQx and dG-C8-PhIP (Toronto Research Chemicals), from peripheral blood samples to determine red or processed meat-induced response. In brief, genomic DNA was extracted from the buffy coat of each peripheral blood sample with a Quick DNA Mini Kit (Zymo Research, Irvine, CA, USA), following the manufacturer's instructions. Isolated DNA (1.5 µg) was digested with a DNA Degradase PlusTM Kit (Zymo Research). Finally, we analyzed dG-C8-MeIQx and dG-C8-PhIP with the same LC/MS/MS system and conditions as for MeIQx and PhIP. Table S2 shows transitions of multiple reaction monitoring (MRM) and conditions for MeIQx, dG-C8- MeIQx, PhIP, and dG-C8-PhIP, including their internal standards, MeIQx-d_3_, dG-C8-MeIQx-d_3_, PhIP-d_3_, and dG-C8-PhIP-d_3_. MassHunter software (Agilent) was used for quantitative data analyses. For relability, most of biomarkers were measured twice.

### Statistical analyses

Shapiro-Wilk W test was used to analyze distributional normality for levels of biomarkers. Due to normality, we used Student's t-test or Mann-Whitney U test for comparison between CRC cases and controls. We also used contingency tables for the categorized biomarkers. To screen associations among biomarkers, we performed Pearson or Spearman rank correlation, and regression analyses. We used multiple regressions to analyze effects of various biomarkers for red or processed meat on CRC. Statistical significance was considered at P < 0.05. JMP ver. 4 (SAS Institute, Cary, NC, USA) was used for all statistical analyses.

## Results

### Characteristics of subjects

Their consumption was 53.4 ± 74.0 g/day for red meat and 1.1 ± 3.7 g/day for processed meat. Table 1 shows characteristics of subjects. CRC patients were more male, tobacco smoking, and alcohol drinking, however, had lower body mass index (BMI) than controls. Although the intake of red or processed meat was not higher in CRC patients than controls, well cooked meat was more preferred by CRC patients than controls, who consumed more vegetables than CRC patients. The cases preferred more well done meat than controls. Interestingly, there were positive associations between two habits, intake of red meat and smoking or alcohol drinking (N = 218; red meat *vs.* smoking, Pearson's r = 0.18, P < 0.01; red meat *vs.* drinking, 0.16 and < 0.05). However, red or processed meat intake did not increase the risk of CRC by interaction with smoking or alcohol drinking (Table 2). Rather, the meat intake showed some tendency to prevent from CRC.

### Biological monitoring

**Figure 1:**
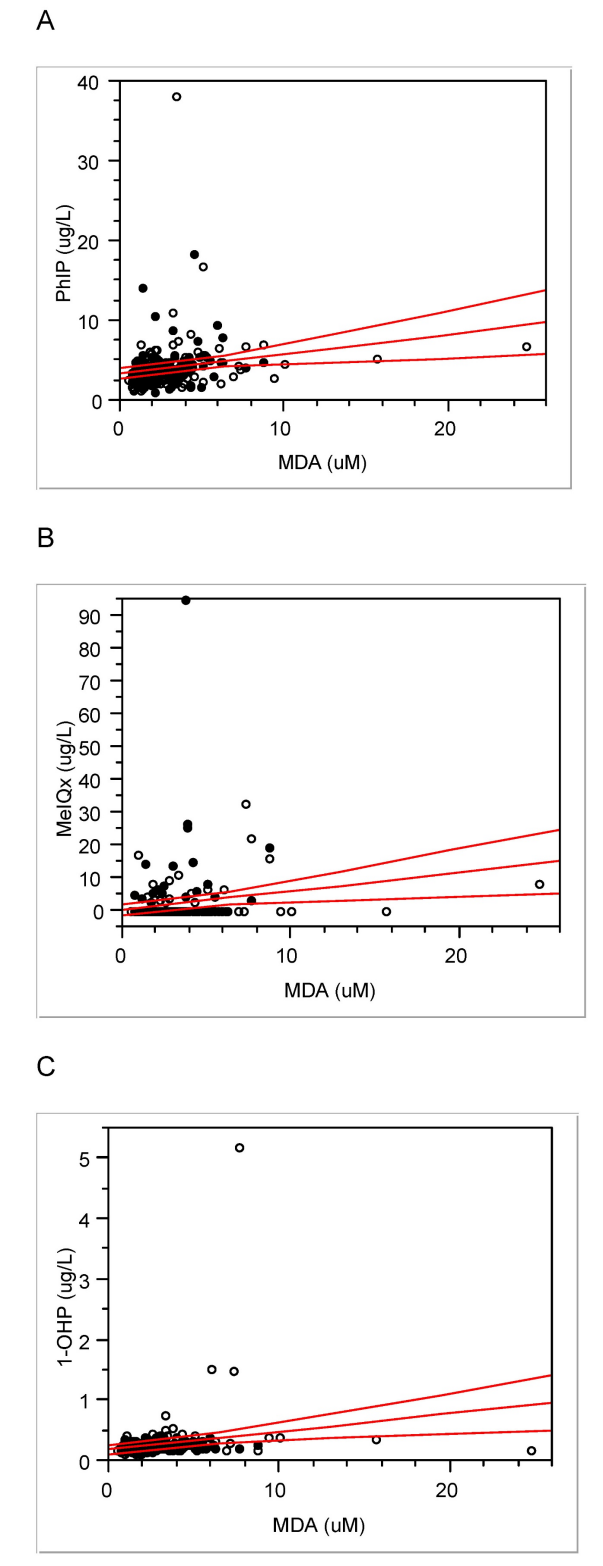
Associations between urinary MDA and PhIP (A), MeIQx (B) or 1-OHP (C); correlation coefficient (r) and P- value for PhIP, respectively, 0.19 and < 0.01; for MeIQx, 0.18 and < 0.01; for 1-OHP, 0.20 and < 0.01; solid line, trend line; dotted line, 95% confidence limits with the confidence curve fit; open circle, controls; closed circle, cases.

Urinary levels of 1-OHP, MeIQx, PhIP, and MDA were 0.28 ± 0.36 (median, 0.24) µg/L, 1.98 ± 0.36 (0.23) ng/L, 4.09 ± 3.19 (3.44) ng/L, and 2.96 ± 2.34 (2.34) µM, respectively. Interestingly, MDA levels were positively associated with the other three exposure biomarkers (Fig. 1; MDA vs. PhIP, Pearson's r = 0.19 and P < 0.01; MDA vs. MeIQx, 0.18 and < 0.01; MDA vs. 1-OHP, 0.20 and < 0.01). As MDA represents oxidative stress or ROS 13, these associations support that PAHs and HCAs induce ROS as a carcinogenic mechanism.

**Figure 2:**
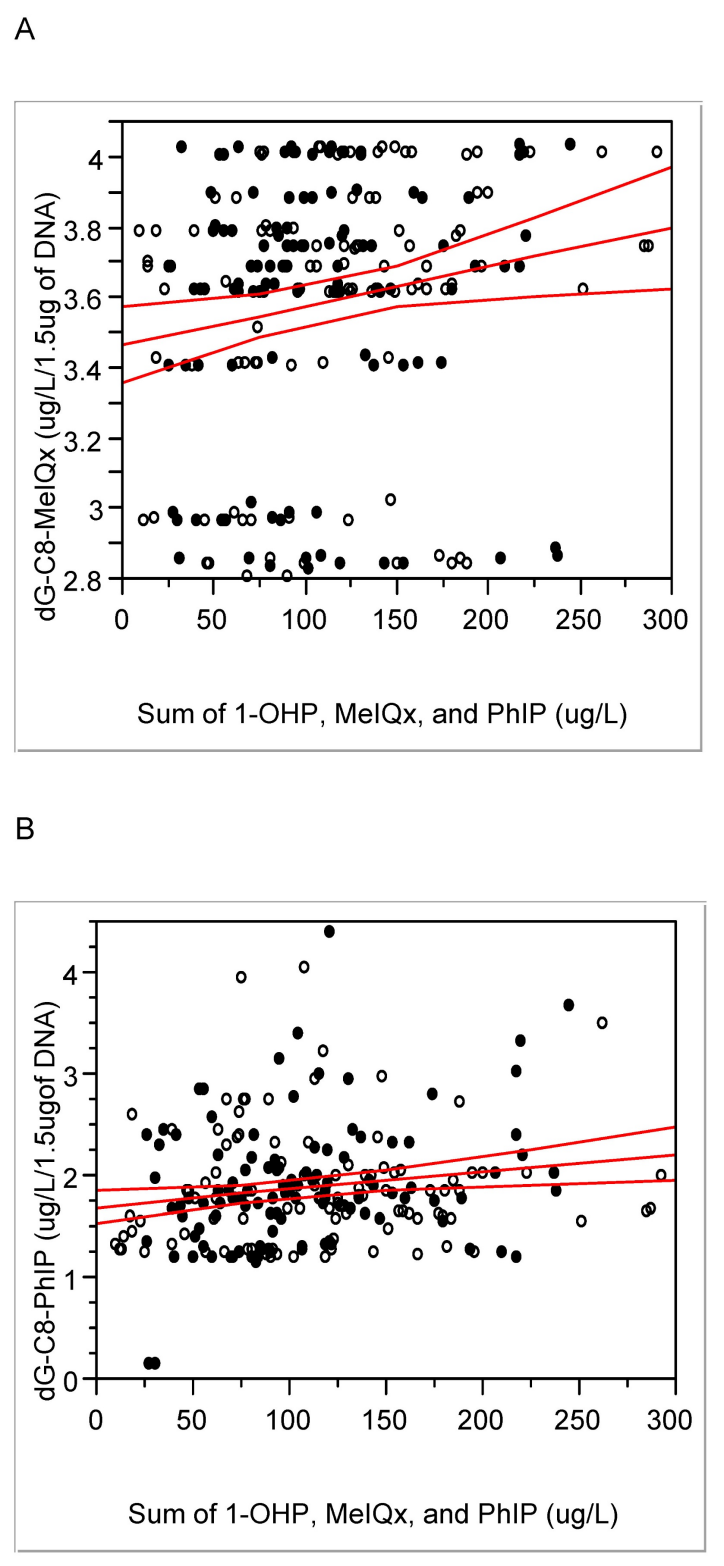
Positive association between integrated exposure and DNA-adducts of MeIQx (A) or PhIP (B): correlation coefficient (r) and P- value for MeIQx-DNA adducts, respectively, 0.19 and 0.004; for PhIP-DNA adducts, 0.14 and 0.04; solid line, trend line; dotted line, 95% confidence limits with the confidence curve fit; open circle, controls; closed circle, cases.

Levels of HCA-DNA adducts, i.e., dG-C8 MeIQx and dG-C8 PhIP in blood, were 3.59 ± 0.38 (median 3.69) and 1.89 ± 0.59 (1.80) µg/L/1.5 µg of DNA, respectively. These two adduct levels were positively associated with each other (r = 0.25, P < 0.01) and positively associated with integrated exposure, i.e., the sum of 1-OHP, MeIQx, and PhIP (Fig. 2). In a case of the cooking method for meat, i.e., degrees of cooked meat, it was not associated with each of exposure biomarker, e.g., urinary 1-OHP, PhIP, or MeIQx (P = 0.12, 0.21, or 0.77, respectively), but associated with dG-C8-MeIQx (0.05 < P < 0.1), with the sum of MeIQx, PhIP, and 1-OHP (P < 0.01), and with oxidative stress biomarkers including MDA and homocysteine (P = 0.01 and P < 0.001, respectively). Thus, the above exposure and response biomarkers were confirmed to reflect consumption of red or processed meat with the integrated exposure or cooking degree.

### Comparison of biomarkers

Among hematological and biomonitoring biomarkers, CRP, an oxidative stress marker 12, 14, and LDL-C levels were higher in CRC patients than in controls. However, blood homocysteine and urinary MDA levels were higher in controls than in CRC patients. In addition, urinary 1-OHP was higher in controls than in CRC patients (Table 3). As the subjects were not matched with sex (Table 1), we also adjusted the results for sex and found a similar trend to those of univariate analyses.

We also investigated food consumption one year prior to diagnosis to avoid food intervention after the diagnosis. To overcome the limitation of a cross-sectional study, we considered DNA-adducts as longitudinal exposure biomarkers for red or processed meat, compared to other exposure biomarkers with short half-lives within a day 15. However, there was no significant difference in the HCA-DNA adducts between CRC patients and controls (Table 3).

### CRC and red or processed meat intake

Considering the results of bivariate associations (Table 1), we re-analyzed effects of red or processed meat intake on CRC. For that, we made three models of multiple regression with the different biomarkers for red or processed meat (Table 3). Model 1 included short term or total exposure biomarkers for the meat. Model 2 was with intake of the meat. Model 3 was with long term exposure biomarkers for the meat. As a result, the risk of CRC was not associated with total exposure (the sum of 1-OHP, MeIQx, and PhIP in µg/g cre), intake of red or processed meat, or HCA-DNA adducts (Table 4). However, vegetable intake consistently showed protective effects on CRC.

When we re-analyzed effects of categorized values for biomarkers and food intake on CRC, e.g., high and low groups, we observed that cases belonged to low exposure groups of 1-OHP, and the sum of 1-OHP, MeIQx, and PhIP, and to the low intake groups of wholegrains or green tea, which have been known protective for CRC 16-17 (Ps < 0.05).

## Discussion

### Characteristics of subjects

Well-known CRC-related factors 18, such as alcohol drinking, tobacco smoking, degree of cooked meat, and vegetable intake, were also associated CRC presence in the present subjects (Table 1). BMI followed the general trend at 1-2 years before CRC diagnosis, i.e., a decrease of BMI in CRC patients 19. Thus, the present subjects showed most of the universal features for CRC 20.

For age and sex, the incidence and mortality of CRC in Korean populations over 65 years old are higher in women than in men, implying that CRC is a major health threat for older women 21. However, sex did not so much affect the CRC presence in this study (Table 3 & 4).

Based on FFQ results, the present subjects were estimated to consume lower levels of red meat (Table 1) than the average daily intake of red meat in Koreans, 69.5 g in *Korea National* Health & Nutrition Examination Survey 21, which is even lower than those of Western countries, e.g., approximately half of U.S.A. 22. In the case of processed meat, such as bacon, hot dogs, and sausage, known to be naturally high in amines 23, the present subjects also consumed less than the estimated usual amount in Koreans (average: 1.10 vs. 4.33 g/day) 22.

### Biological monitoring

Compared to our previous pilot study 6, the present enlarged study showed similar or somewhat high levels of urinary 1-OHP, PhIP, and MDA, i.e., median, 0.14 vs. 0.24 µg/L, 3.44 vs. 3.44 µg/L, 1.79 vs. 2.34 mM, respectively. However, urinary levels of MeIQx in the present subjects were lower than those in our previous pilot study, i.e., 1.98 vs. 13.51 ng/L 6. As our previous study was the first in the world to analyze human HCA-adducts in blood 6, we compared the present DNA adduct levels to the previous ones and found they were similar to each other. The trend that the higher levels of dG-C8 MeIQx in CRC patients than in controls, which was found in the previous study 6, was not observed in the present study (Table 2).

When the present HCA-DNA adduct levels were converted into pmol/mg of dG, they were approx. hundreds pmol adducts /mg of dG and seem to be relatively higher than other xenobiotics-DNA adducts, e.g., DNA-adducts with 4-hydroxy-1-(3-pyridyl)1-bubutanone, an analog of nicotinic acid for tobacco smoking, in oral cells, 12 pmol adducts /mg of DNA in smokers 6, 24. In addition, the property of biomarkers for red or processed meat intake was confirmed by positive associations between the degree of cooked meat and levels of dG-C8-MeIQx (Pearson's r = 0.14 and P < 0.05) and between HCA-DNA adducts and the sum of exposure, such as 1-OHP, HCAs, PHIP, and MeIQx (Fig. 2; DNA-adducts of MeIQx *vs.* integrated exposure, Pearson's r = 0.19 and P < 0.01; DNA-adducts of PhIP-DNA *vs.* integrated exposure, 0.14 and < 0.05). Thus, we further studied differences in lifestyle, such as food intake pattern or degree of meat cooking that might affect exposure or response biomarkers.

### Comparison of biomarkers

As PAHs are present in meats at a high temperature, they showed strong associations with CRC risk in population studies 25. We measured urinary 1-OHP as internal dose of PAH exposure and the levels of 1-OHP was 0.07-5.35 (median, 0.27) µg/g creatinine, which is similar to current levels of Korean National Environmental Health Survey data (50 percentile, 0.20 µg/g creatinine) 26. However, we found that urinary 1-OHP levels were not associated with intake of red meat or processed meat (p = 0.51 or 0.27, respectively). The controls even showed higher levels of 1-OHP than CRC cases (Table 3). As the half-life of parent chemical of 1-OHP, pyrene, is short in humans, approx. 3 hrs 25, 27, urinary 1-OHP may reflect current exposure to PAHs. The oxidative stress levels from urinary MDA and blood homocysteine were strongly associated with urinary 1-OHP levels and theses biomarkers were also higher in controls than cases (Table 3). Thus, the people free CRC with high levels of 1-OHP, MDA, or homocysteine should be carefully monitored for preemptive prevention of CRC, due to the potential risks of oxidative stress and long latent period of CRC.

Oxidative stress, ROS, or repeated inflammation have been emphasized as potential risks or toxic mechanisms of CRC from consuming red or processed meat 8, 28-29. For example, levels of MDA, a biomarker for oxidative stress, were higher in gastrointestinal contents and colonic tissues of rats fed beef diets than those before digestion 30. In addition, oxidative stress and mitochondrial dysfunction are known as ones of the toxic mechanisms of HCAs 31. We also found that urinary MDA levels were positively associated with those of PhIP, MeIQx and 1-OHP (Fig. 1) and the preference to well-done meat (r = 0.17, p = 0.01). Thus, theses associations support PAHs or HCA -induced oxidative stress as a mechanism of carcinogenesis of CRC. In addition, two oxidative stress biomarkers, MDA and homocysteine levels, were borderline-significantly associated with each other in the present study (r = 0.13, p = 0.05). However, they were higher in the controls than the cases, contrary to our expectation (Table 3). Thus, these oxidative stress levels seem to be not enough for CRC prevalence in the present subjects.

Considering the time difference between current biomonitoring and diet style one year ago, we can estimate the exposure biomarkers, i.e., MDA, PhIP, MeIQx, and 1-OHP, reflect current exposure to red or processed meat. Thus, more chronic or longitudinal exposure biomarkers are needed to monitor effects of red or processed meat than the above biomarkers. Thus, HCA-DNA adducts, which have longer half-lives than 1-OHP, PHIP, or MeIQx 32 have been used to elucidate genotoxic mechanisms of red or processed meat, to avoid limitation of cross-sectional studies, and to estimate summation of recent and chronic exposures 15. In the present study, HCA-DNA adducts were positively associated with the sum of 1-OHP, MeIQx, and PhIP (Fig. 2). Thus, DNA-adducts can be potential and desirable response biomarkers for long exposure.

### CRC and red or processed meat intake

We used these various biomarkers as well as the amount of meat consumed to clarify the effect of meat consumption on CRC. However, there were little effects of the intake of red or processed meat or various biomarkers for meat on CRC by multiple analyses (Table 4) or bivariate analyses (Table 1). A recent epidemiolocal study showed only weak associations between overall red meat and processed meat intake and CRC risk in Jewish and Arabs 15, 33, who less consumed meat than most of Europeans or north Americans, however, more consumed meat than Koreans (beef and veal in Israel vs. Korea, average 24.1 vs 12.4 kg capita/year) and support the present results 22. In addition, a review for dietary red meat on CRC in Asians suggested that multiple factors including fruit and vegetable intake, alcohol consumption, smoking, obesity, or stress should be considered when evaluating the risk of CRC rather than only considering meat intake 34. Moreover, a current systematic review of 69 studies showed red meat or total red and processed meat were risky for CRC not in Eastern, 1.01 (0.91-1.13) or 1.04 (0.91-1.18) as RR (95% CI), respectively, but in Western people, 1.12 (1.04-1.19) or 1.15 (1.07-1.23), respectively 35. Therefore, the effects of red or processed meat intake on CRC seem to be relatively weak, compared to integration effects on CRC in Asians, who consume less meat than Western people.

Considering the advantage of biological monitoring to clarify various causes of CRC 36, we conclude that red or processed meat may induce oxidative stress, however, present intake of the meat and the intake-related oxidative stress may not affect CRC prevalence among the Korean population, who consume less meat than Westerners.

## Supplementary Material

Supplementary figures and tables.

## Figures and Tables

**Table 1:** Characteristics of the subjects in lifestyle

Variables	Control		CRC			P -value				
	Mean	STD ^c^	Mean	STD						
Age (yrs)	63.28	14.13	65.59		12.61			0.12		
Sex [N of male (%)]	46 (42.20)	-	70 (64.22)		-			0.04		
Body weight (kg)	64.21	12.56	61.56	12.43			0.12			
BMI (kg/m^2^)	24.57	3.59	23.25	3.71			< 0.01			
Tobacco [N (%)]							< 0.01			
Never smoker	81 (74.31)		52 (47.71)							
Ex-smoker	20 (18.35)		45 (41.28)							
Smoker	8 (7.34)		12 (11.01)							
Alcohol [N (%)]							*<* 0.01			
Never drinker	71 (65.14)		39 (35.78)							
Ex-drinker	19 (17.43)		42 (38.53)							
Drinker	19 (17.43)		28 (25.69)							
Red meat (g/day)	54.74	92.79	52.01	48.89			0.78			
Processed meat (g/day)	1.18	4.23	1.02	3.11			0.75			
Degree of cooked meat [N (%)]^a^							0.02			
Rare	1 (0.92)		1 (0.92)							
Medium rare	7 (6.42)		0 (0)							
Medium	12 (11.01)		15 (13.76)							
Medium well	23 (21.10)		17 (15.60)							
Well	65 (59.63)		72 (66.06)							
Vegetable [N (%)]^b^							0.03			
< one meal	15 (13.76)		31 (28.44)							
One meal	10 (9.17)		10 (9.17)							
Two meals	33 (30.28)		20 (18.35)							
Every meal	51 (46.79)		48 (44.04)							
Fruit [N (%)]							0.14			
< one meal	62 (56.97)		77 (70.65)							
One meal	38 (34.86)		27 (24.77)							
Two meals	5 (4.59)		2 (1.83)							
Every meal	4 (3.67)		3 (2.75)							
Dietary inflammatory index	3.37	2.02	3.67	2.02			0.27			
Exercise [N (%)]^c^							0.82			
1-2 times/week	22 (38.60)		20 (39.22)							
3-4 times/week	22 (38.60)		16 (31.37)							
5-6 times/week	8 (14.04)		10 (19.61)							
Every day	5 (8.77)		5 (9.80)							
										

N of N/A, ^a^5;^ b^20; ^c^110 The definition of ex-smoker followed that of NHIS/ CDC, i.e., an adult who has smoked at least 100 cigarettes in his or her lifetime but who had quit smoking at the time of interview. The definition of ex-drinker followed that of WHO, i.e., adults who did not consume alcohol in the last 12 months, but who did previously do that

**Table 2:** Interactions between intake of red or processed meat and smoking or alcohol on CRC

Meat Intake	Alcohol Never	Drinker	Tobacco Never	Smokers
Red meat^a^				
Low	1	1.83-9.97 (4.18)	1	1.37-7.70 (3.17)
High	0.43-2.13 (0.96)	1.45-5.89 (2.89)	0.47-1.89 (0.94)	1.45-6.52 (3.03)
				
Processed meat^b^				
Low	1	1.63-6.11 (3.12)	1	1.48-5.89 (2.92)
High	0.13-1.20 (0.43)	0.73-4.32 (1.74)	0.25-1.36 (0.60)	1.13-6.42 (2.62)

Data show odds ratios (OR) with 95% confidence interval, low-high (average) ^a^ high > median, 39.5g/day; low ≤ median ^b^ high > median, 0.00 kg/day; low ≤ median

**Table 3:** Comparison of biomarkers between the controls and the CRC patients

Biomarkers	Control		CRC		P -value^ b^	P -value^c^
	Mean	STD	Mean	STD		
Hematological						
AST (U)	12.77	6.67	25.41	12.60	0.09	0.24
ALT (U)	11.09	9.92	21.90	13.83	0.49	0.57
CRP (mg/dL)	0.34	1.14	1.33	2.92	0.00	0.00
TC (mg/dL)	172.27	48.33	174.95	43.35	0.35	0.34
TG (mg/dL)	134.72	74.63	118.54	76.10	0.06	0.08
LDL-C (mg/dL)	89.63	32.17	101.80	36.77	0.01	0.00
HDL-C (mg/dL)	49.55	17.80	50.00	16.73	0.42	0.26
Homocysteine (µM)	11.02	4.90	8.37	4.14	0.00	0.00
Biomonitoring						
Urinary MDA (µM/g Cre)	3.99	3.38	2.92	1.61	0.04	0.01
Urinary 1-OHP (µg/g Cre)	0.43	0.55	0.32	0.22	<0.01	0.08
Urinary MeIQx (ng/g Cre)	1.92	5.11	2.92	12.24	0.74	0.51
Urinary PhIP (ng/g Cre)	5.47	4.76	5.45	4.92	0.45	0.82
dG-C8 MeIQx ^a^	3.59	0.37	3.58	0.38	0.89	0.10
dG-C8 PhIP^ a^	1.87	0.56	1.91	0.63	0.35	0.12

^a^ (ppt/ 1.5 µg of DNA) ^b^univariate comparison between the levels of each biomarker and CRC presence ^c^comparison between the levels of each biomarker and CRC presence, adjusted for sex

**Table 4:** Lack of association between CRC and red or processed meat intake, adjusted for other risks

	Model 1		Model 2		Model 3	
Variable	Regression coefficient	P-value	Regression coefficient	P-value	Regression coefficient	P-value
Sex^a^	-0.07	0.38	-0.08	0.36	-0.09	0.31
BMI	-0.03	0.00	-0.03	0.00	-0.03	0.00
Alcohol^b^	0.09	0.06	0.09	0.06	0.00	0.36
Tobacco^c^	0.08	0.21	0.08	0.24	0.11	0.08
Vegetable	-0.22	0.02	-0.05	0.02	-0.05	0.01
Degree of cooked meat^d^	0.01	0.32	0.00	0.29	0.00	0.35
Sum of 1-OHP, MeIQx, or PhIP (µg/g cre)	0.00	0.40	-	-	-	-
Intake of red meat	-	-	-0.03	0.52	-	-
Intake of processed meat	-	-	-0.12	0.89	-	-
dG-C8-MeIQx (µg/l/1.5µgDNA)					0.02	0.78
dG-C8-PhIP (µg/l/1.5µg DNA)					0.03	0.63
R^2^	0.15		0.15		0.14	

Note: values of multiple regression: Y= 0 (control) or 1 (CRC); For x values, ^a^man = 1, woman = 2; ^b^non-drinker = 1, drinker = 2; ^c^non- smoker = 1, smoker = 2; ^d^scale from 1 (rare) to 5 (well-done).
