# Supplementary material for: In-depth Biological Monitoring to Estimate Effects of Red or Processed Meat on Colorectal Cancer
Source: J Cancer. 2026 Jan 14;17(2):395–403. doi: 10.7150/jca.126466 (PMC12825417; doi:10.7150/jca.126466)
Supplement: Supplementary file 1 — Supplementary figures and tables. [file jcav17p0395s1.pdf]

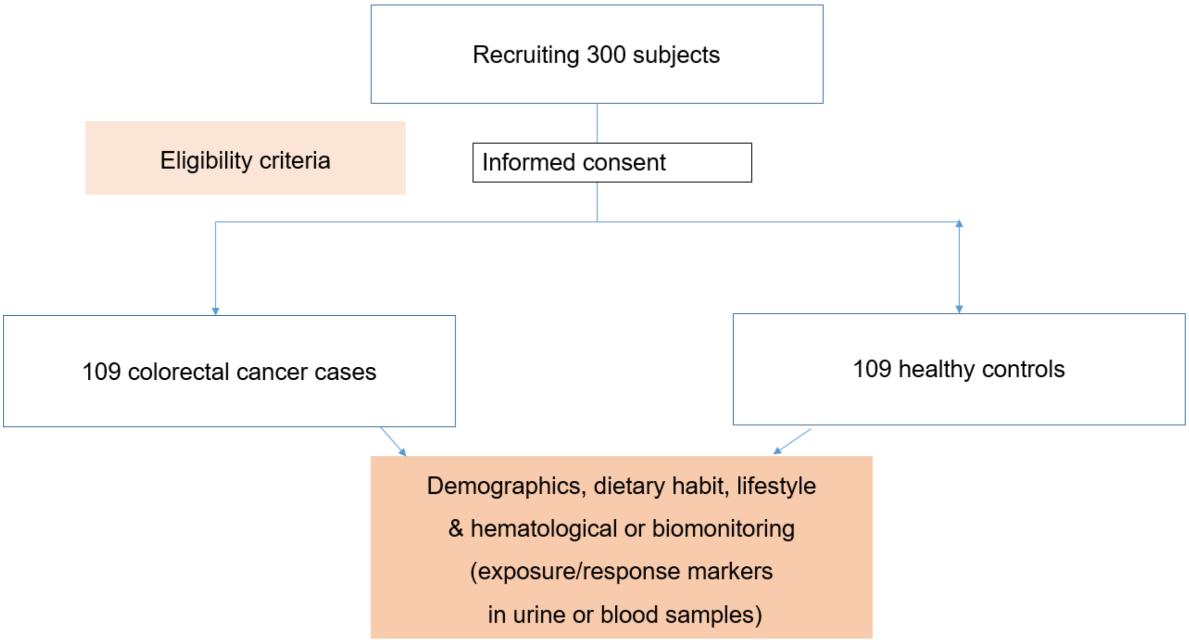

S Fig. 1. Study flow

S Table 1. Enrollment requirements

Case		Control	
Inclusion	Exclusion	Inclusion	Exclusion
1. Patients newly di agnosed (within 2 wee ks) with CRC	1. Patients with any other history of cancer, polyps, or inflammatory bowel disease	1. Participants withou t any history of cancer, polyps, or inflammator y bowel disease	1. Participants requirin g active treatment or h ospitalization
2. Age over 20 years o ld	2. Patients treated with anti-cancer treatment, including surgery, radiation or any medication for CRC	2. Age over 20 years o ld	2. Participants involve d in any intervention f or diet modification
3. Patients ready for th e first anticancer treat ment for CRC	3. Patients involved in any intervention for diet modification	3. Participants who un derstand the questionn aire	3. Participants with fa mily history of CRC
4. Patients who unders tand the questionnaire		4. Participants who agr eed informed consent	4. Participants needed diet modification with diseases in kidney, hea rt, endocrine, liver, or gastro-intestine
5. Patients who agreed informed consent	4. Foreigners with diff erent diet from Korean s		5. Foreigners with diff erent diet from Korean s

S Table 2. MRM conditions and fragmentation patterns from LC/MS/MS analysis

Compound	Precursor ion (m/z)	Product ion (m/z)	Collision energy (eV)	Dwell time (msec)
MeIQx	214.1	199	30	20
		131	45	20
MeIQx-d3	217.1	199	34	20
		131	42	20
dG-C8 MeIQx	479.1	363.1	17	20
		299.1	64	20
dG-C8 MeIQx-d3	482.1	366.1	17	20
PhIP	225.1	210	34	20
		140	45	20
PhIP-d3	228.1	210	34	20
		140	45	20
dG-C8 PhIP	490.1	374.1	20	20
		250.1	60	20
dG-C8 PhIP-d3	493.1	377.1	25	20

a.

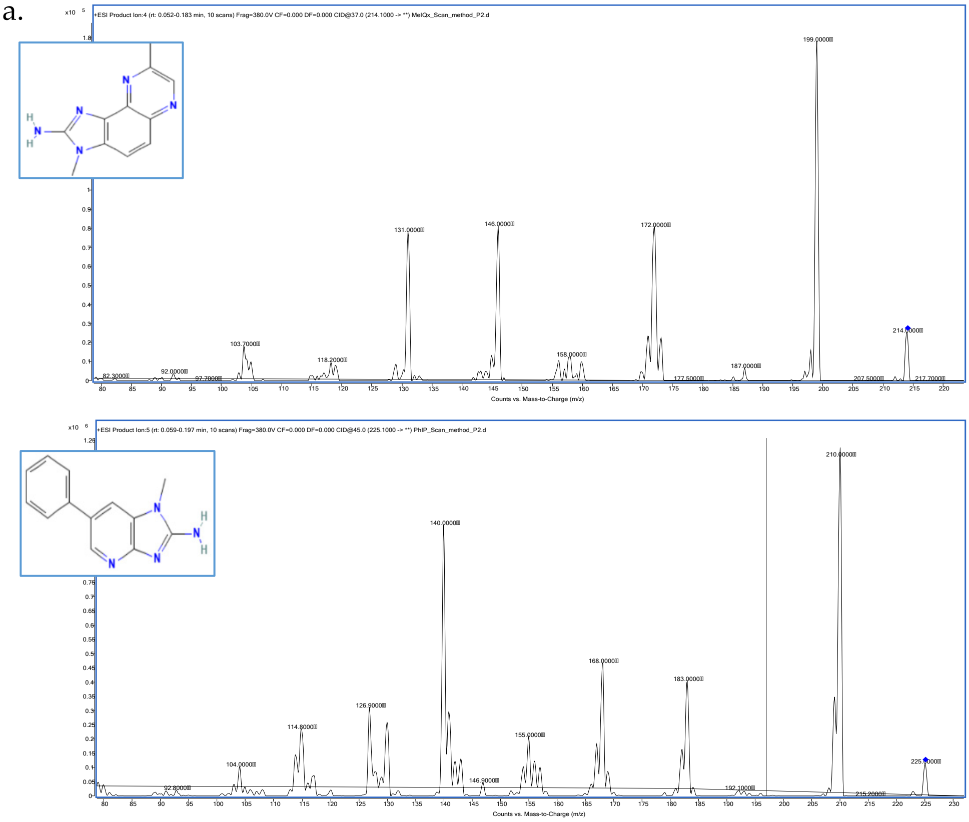

b.

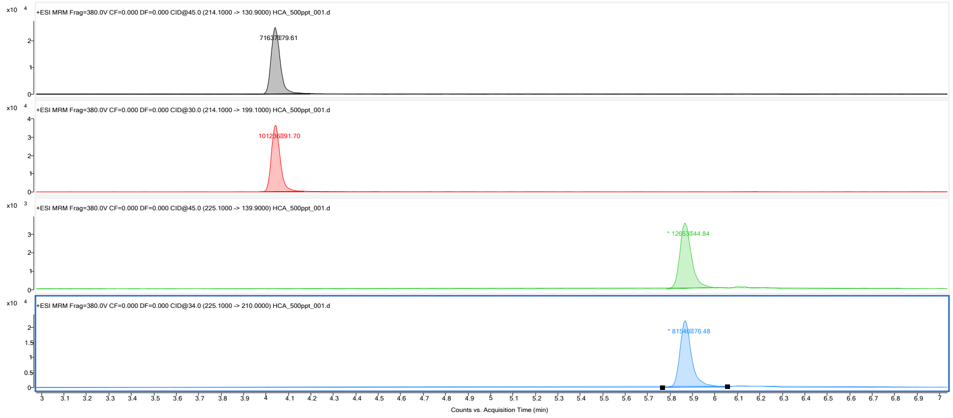

S Fig.2. Method development for LC/MS/MS to analyze urinary MeIQx and PhIP a) MRM spectra of urinary MeIQx and PhIP displaying the correspondent structures; b) MRM chromatograms of the major molecular ions for the quantification and qualification of MeIQx and PhIP, respectively (in order from the top)

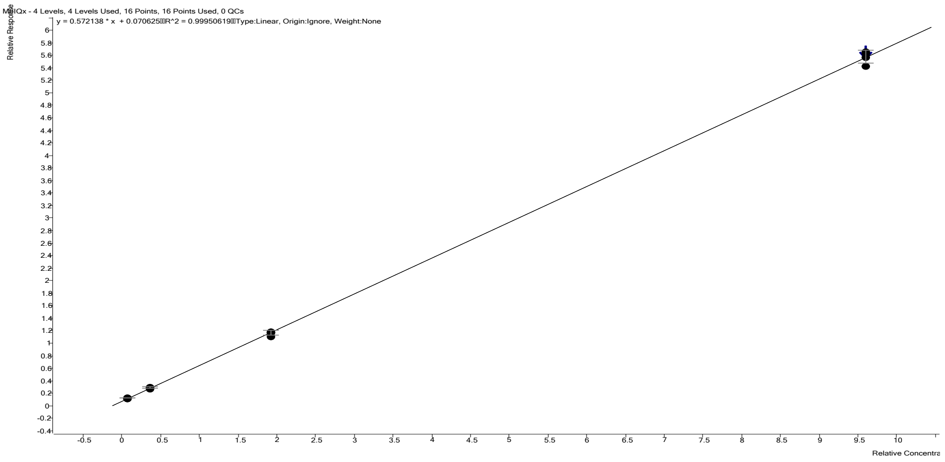

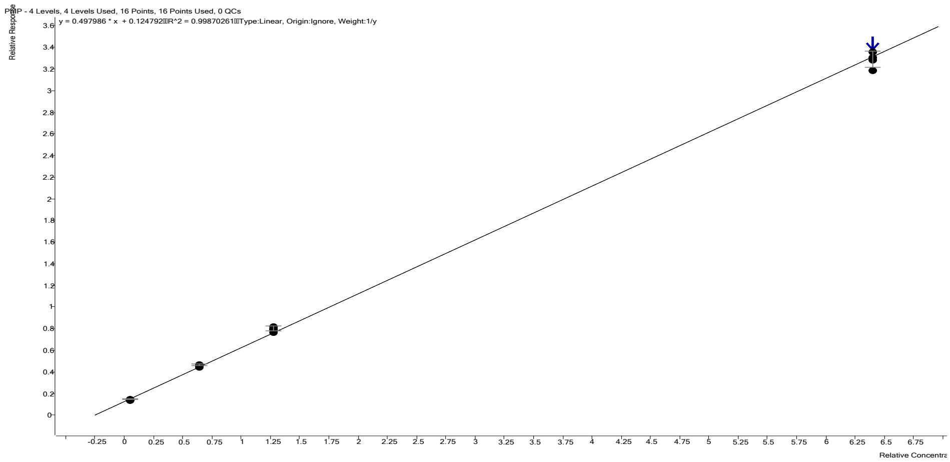

S Fig. 3. Calibration curves of PhIP (top) and MeIQx (bottom) reporting coefficients of regression (R^2) higher than 0.998″
